# Efficacy of sucralfate ointment in the prevention of acute proctitis in cancer patients: A randomized controlled clinical trial

**DOI:** 10.22088/cjim.11.4.410

**Published:** 2020

**Authors:** Sara Saei, Adeleh Sahebnasagh, Arash Ghasemi, Jafar Akbari, Abbas Alipour, Hossein Lashkardoost, Ali Yaghobi Joybari, Farid Nejad Dadgar, Shahram Ala, Ebrahim Salehifar

**Affiliations:** 1 Student Research Committee, Faculty of Pharmacy, Mazandaran University of Medical Sciences, Sari, Iran.; 2 Clinical Research Center, Department of Internal Medicine, Faculty of Medicine, North Khorasan University of Medical Sciences, Bojnurd, Iran.; 3 Emam Khomeini Hospital, Mazandaran University of Medical Sciences, Sari, Iran.; 4 Pharmaceutical Research Center, Faculty of Pharmacy, Mazandaran University of Medical Sciences, Sari, Iran.; 5 Department of Epidemiology, Faculty of Medicine, Community Medicine Department, Mazandaran University of Medical Sciences, Sari, Iran; 6 School of Public Health, North Khorasan University of Medical Sciences, Bojnurd, Iran; 7 Pharmaceutical Research Center, Faculty of Pharmacy, Mazandaran University of Medical Sciences, Sari, Iran; 8 Gastrointestinal Cancer Research Center, Faculty of Pharmacy, Mazandaran University of Medical Sciences, Sari, Iran

**Keywords:** Radiotherapy, Sucralfate, Acute Proctitis, Pelvic, Ointment

## Abstract

**Background:**

Acute radiation proctitis (ARP) is a usual adverse effect in patients undergoing pelvic radiotherapy. The symptoms include diarrhea, rectal blood or mucus discharge, fecal urgency and tenesmus with pain. Sucralfate, an aluminum-based salt of sucrose octasulfate, is a cytoprotective agent that forms a coating barrier at injured sites by adhering to mucoproteins. It has been used in topical management of a wide variety of local lesion. This study was designed to evaluate the preventive effect of rectal sucralfate on acute radiotherapy induced proctitis.

**Methods:**

Seven percent sucralfate ointment was prepared for topical use. Drug quantification, chemical stability and microbial limit tests were performed carefully. In this randomized double blind placebo controlled trial, fifty-seven patients with pelvic malignancies undergoing radiotherapy were allocated to receive either 1 g of sucralfate or 1 g of placebo, given as a twice daily ointment, one day before and during radiotherapy for six weeks. The eligible patients were evaluated based on RTOG acute toxicity criteria and the following ARP symptoms weekly: rectal hemorrhage, diarrhea, rectal pain, and fecal urgency. The influence of symptoms on lifestyle was also recorded weekly.

**Results:**

Acute proctitis was significantly less prevalent in patients in the sucralfate group. The incidence of rectal bleeding (P=0.003), diarrhea (P=0.002), rectal pain (P=<0.001) and fecal urgency (P=0.002) was significantly less common in the sucralfate group. No statistical significant difference was observed for radiotherapy induced cystitis in the placebo and sucralfate groups (P=0.27).

**Conclusion:**

This study suggests that sucralfate7% ointment reduces the incidence of symptoms associated with acute radiation proctitis.

Acute radiation proctitis is a common adverse effect of radiotherapy to the pelvic. There is no widely accepted prophylaxis for proctitis. Sucralfate has cytoprotective effects by forming a protective barrier in a wide variety of wounds as topical preparations. This study was designed to evaluate prospectively the rectal sucralfate on acute radiotherapy-induced proctitis. Radiotherapy (RT) is extensively used in the treatment of several kinds of malignancies including pelvic malignancies. Radiation-induced complications occur in most patients athwart the advent of conformal techniques (1). Due to the short cell cycle time of the epithelial cells of the intestine, the intestinal mucosa is sensitive to radiation and acute proctitis is a common adverse effect following this therapy (2-5). Changes in the normal colonic bacterial flora may also play a role in the incidence of acute proctitis (4). Acute radiation proctitis is a common adverse effect of radiotherapy to the pelvic. There is no widely accepted prophylaxis for proctitis. Sucralfate has cytoprotective effects by forming a protective barrier in a wide variety of wounds as topical preparations.

This study was designed to evaluate prospectively the rectal sucralfate on acute radiotherapy-induced proctitis. Radiotherapy (RT) is extensively used in the treatment of several kinds of malignancies including pelvic malignancies. Radiation-induced complications occur in most patients athwart the advent of conformal techniques (1). Due to the short cell cycle time of the epithelial cells of the intestine, the intestinal mucosa is sensitive to radiation and acute proctitis is a common adverse effect following this therapy (2-5). Changes in the normal colonic bacterial flora may also play a role in the incidence of acute proctitis (4). The adverse effects may appear during or shortly after irradiation (6) and the main symptoms include rectal pain, rectal hemorrhage, diarrhea and fecal urgency (7). Although the symptoms are mostly self-limited, the occurrence of rectal injury could further decrease the patient’s quality of life (8). Conventionally, oral and rectal medications including glucocorticoids, bile acid sequestrants, anti-diarrheal medicines, sulfasalazine, and antibiotics have been used as preventive or treatment measure. New radiation techniques to sculpt precise dose to affected tissues are not available in all institutions (4).Yet, little have they proven to be of benefit and have been associated with high adverse effects (9-11). At the moment, there is no standard approach for the prevention and treatment of acute radiation proctitis (12). Therefore, investigating the novel ways for prevention of rectal injury is essential. Sucralfate, a basic aluminum salt of sucrose octasulfate, provides a coating barrier at the injured sites (13, 14). 

Sucralfate has been topically beneficial in treating chronic venous ulcer (15), perianal skin irritation (16), pain reduction after hemorrhoidectomy (17), fistulotomy wounds (18) and second and third degree burns (19). Oral sucralfate has also been beneficial in the prevention of acute proctitis (6). However, even the strongest evidence for the use of sucralfate in acute radiation proctitis has been discredited by some limitations, such as uncertain allocation concealment, short follow-up period and lack of explanation for patients’ lost to follow-up (20). Thus, the beneficial effects of topical sucralfate and its possible side effects in pelvic injuries should be examined during a longer period of time. Hopefully, higher doses of this topical agent, concentrating in the affected area, will open some new ways in alleviating patient suffering. This double blind randomized study aimed to evaluate the positive effects of topical formulation of sucralfate 7% in the prevention of radiotherapy induced proctitis compared with placebo. In addition to the developement of proctitis symptoms as our primary endpoint, we evaluated the lifestyle impact of the symptoms, as secondary endpoint.

## Methods

***Study design and ethical considerations: ***This double-blind, placebo controlled, randomized trial evaluated the effectiveness of topical sucralfate in the prevention of acute-radiation proctitis in pelvic radiaotherapy. The Ethics committee of Mazandaran University of Medical Sciences approved the study (IR.MAZUMS.REC.94-1743). Besides, it was registered and approved in the registry of clinical trials (IRCT201606042027N7). CONSORT checklist was completed for reporting parallel group randomized trials (Supplementary File 1).

***Preparation of the ointments: ***The incorporation method was used to prepare sucralfate ointments (7% w/w). Liquid paraffin and petrolatum were used as the levigation agent and base, respectively. The same method was used to prepare the placebo ointment, containing liquid paraffin as the levigation agent and petrolatum as the base only. Similar tubes, each capable of 30 grams of the ointment were filled. A six-digit code was labeled on each tube. The eligible cases, the physicians, the healthcare team and the data collectors were blind to the allocated random codes until the end of the study. Drug quantification was done based on titration of aluminum according to the United States Pharmacopeia (21). Besides, microbial limit tests and physicochemical stability of the ointment were performed by the accelerated method in 50, 60, 70 and 80°C. Sterile preparation and formulation of the ointments and quantification and sterility tests were done in a pharmaceutical laboratory in pharmacy school under the direct supervision of a pharmaceutical specialist.

***Settings and Patients: ***The study was conducted in two radiation oncology centers affiliated to Islamic Azad University of Medical Sciences (Tehran) and Mazandaran University of Medical Sciences (Sari) and during a one-year period, patients aged 18 and above who were scheduled to undergo pelvic radiotherapy due to pelvic malignancies were evaluated for eligibility. Patients with active infections, inflammatory bowel disease, previous rectal surgery, hemorrhoids, anal incontinence, anorectal stenosis and fistula, pregnancy or breast feeding, women of child-bearing age with inadequate contraception, allergic to any ingredients of the ointments or proctitis due to previous radiotherapy did not meet entry requirements for enrollment in the trial. 

**Fig 1:**
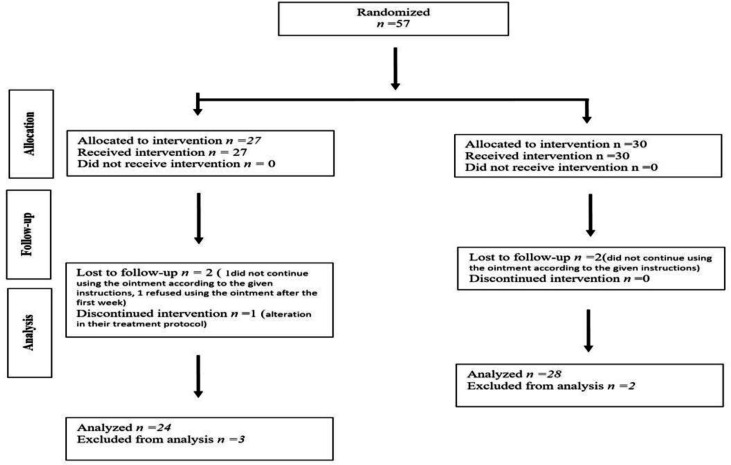
Flow chart of the study

***Calculation of Sample Size and Randomization: ***Since acute radiation proctitis symptoms have a high prevalence, for a power of 0.8, significance level of 0.05, and rate of 10% lost to follow-up, we calculated that 57 patients would be enough for detecting a significant decrease in the symptoms of acute radiation proctitis. Permuted block randomization method was applied for assigning the patients with inclusion criteria into two arms of the study. We used blocks of four. The principal investigator gave a six-digit number to prepared oitments. At the end, the head master of the study decoded the consumed topical preparation. 

***Study intervention: ***Patients eligible for the study prospectively received either a 7% sucralfate or placebo topical preparations. The sucralfate and placebo ointment tubes looked exactly the same. Patients were instructed to use 1 gram of the ointment twice daily one day before and during radiotherapy for six weeks. A paper guidance was handed to each patient along with the ointment for proper method of applying the ointment.

***Outcome measurement: ***Evaluation of the patients was performed a day before and then weekly during radiotherapy for six weeks. Questionnaires on demographic and clinical details of each patient, including cancer diagnosis, co-morbidity, previous treatments and the radiation dose were filled out by the investigators. Details on four main signs of acute proctitis including rectal pain, rectal bleeding or mucus discharge, stool consistency and fecal urgency were questioned and written down based on the Common Terminology Criteria for Adverse Events (CTCAE) 4.03 in the questionnaires (22). Based on their severity, a score between 0-4 was allocated to each sign (0= not present; 1= minor signs, no medicinal intervention required; 2= moderate signs, requiring analgesic/anticholinergic medicine; 3=significant signs; 4= causing significant discomfort, requiring rest or hospitalization). The scores of each sign were added together to reach the total score, that would be a score between 0-16. The influence of symptoms on lifestyle was also recorded based on a questionnaire which grades from 0 (symptoms have no effect on daily routine) to 4 (severe symptoms that the patient is afraid to leave home) (23, 24). 

***Statistical analyses: ***All statistical analysis was conducted using SSPS software Version 19. For the comparison of qualitative variables in two groups of the study, chi-square test and Fisher's exact test were applied to compare. To analyze quantitative variables, Mann-Whitney test was used. P<0.05 were considered to have significant difference statistically.

## Results

Flowchart of the study is displayed in figure 1. During the study, 120 patients completed a cycle of pelvic radiation therapy and they have evaluated for enrollment eligibility. A total of 57 patients were registered to participate in this trial, 27 patients in the sucralfate group and 30 in the placebo group. The participating population consisted of 24 women and 33 men, with a median age of 59.96 years old. Five patients were withdrawn from the study before completion of the trial. Three patients did not follow protocol (1 patient in the sucralfate group and 2 patients in the placebo group), as they did not continue using the ointment according to the given instructions. One patient in the sucralfate group refused to participate in the trial after the second visit. Also, 1 patient from the sucralfate group was excluded because of alteration in their treatment protocol. Table 1 shows a comparison of demographic and clinical features of patients in the two arms of the study. There were no significant differences between the two groups regarding age (P=0.69), sex (P=0.07), BMI (P=0.59), radiation dose in each session (P=0.16), total radiation dose (P=0.25) and cancer type (P=0.30). 

**Table 1:** Baseline Characteristics of Patients

	**Group (mean ± standard deviation/frequency)**	
	**Sucralfate (n=27)**	**Placebo (n=30)**
Age, year	58.33 ± 16.97	59.97±13.70
Sex, F/M	15/12	9/21
BMI	26.00±5.04	25.41±3.14
Radiation Dose (each session) CGy	181.56±14.90	186.133±9.20
Total Radiation Dose	5177.78±1321.052	5542.00±1027.05
Cancer Type		
Bladder	n = 3	n =2
Cervix	n =2	n =4
Endometrial	n =4	n =3
Prostate	n =4	n =11
Other	n=14	n=10

**Figure 2:**
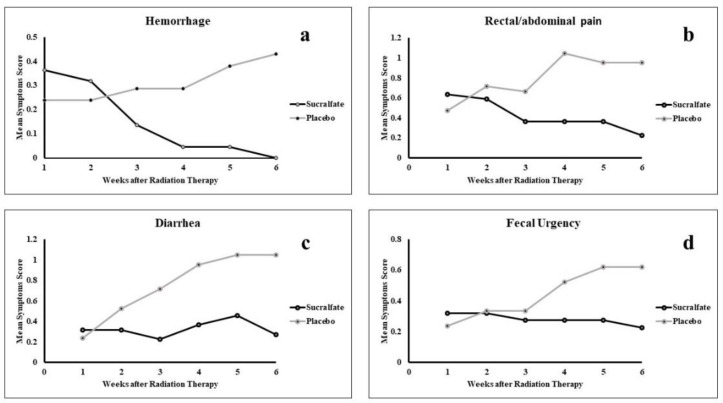
Trend of changes of clinical manifestations of proctitis during 6 weeks of follow-up

Table 2 shows the average scores of clinical manifestations of rectal hemorrhage, diarrhea, rectal pain, fecal urgency and total clinical manifestations over time. Figures 2 and 3 show the outcomes by applying the sucralfate or placebo ointments over the six weeks of the study. 

There was a significant difference in rectal hemorrhage between the sucralfate and placebo group (P=0.003). The average scores of rectal bleeding in the sucralfate group significantly decreased by 0.36 ± 0.66 over the six weeks of the study, and complete cessation of rectal bleeding was observed at the end of week six. While it had an upward trend in the placebo group, from 0.24±0.54 at week 1 to 0.42±0.50 at the end of week six. The average scores of rectal pain followed a downward trend during the time of the study, decreasing from 0.64±0.73 in week 1 to 0.23±0.53 at end of week six. Whereas, the placebo group keeps an upward trend despite minor fluctuations, rising from 0.48±0.68 at week 1 to 0.95±0.80 at week 6 (P<0.001). Starting from 0.32±0.57 at week 1, a slight overall decline in the trend of average scores for diarrhea in the sucralfate group was observed, ending with 0.27±0.45 in week 6. Although fluctuations were observed in the trend of the sucralfate group, the difference between the two arms of the study remains statistically significant (P=0.002). The difference of fecal urgency was also notable in the sucralfate and the placebo group (P=0.002). The trend of fecal urgency drops from 0.32±0.57 to 0.227±0.43 and goes up from 0.24±0.54 to 0.62±0.59 over the study period for the sucralfate and placebo group respectively. The average scores of total clinical manifestations over time were remarkable between the two groups (p<0.001). Table 3 shows the average grades of the severity of the symptoms by the RTOG scores. The differences of cystitis (P=0.27) and proctitis (P=0.76) between the sucralfate and placebo group were statistically insignificant. However, there was a significant difference observed for RTOG diarrhea (P=0.002). The total RTOG scores were different in the two arms of the study (p<0.001). Quality of life (QOL), depression and anxiety were evaluated as secondary endpoints. The quality of life of patients in the sucralfate group significantly improved comparing to the placebo group (P=0.002). Improvements in the depression states of the sucralfate group were observed (p<0.001), however the anxiety state of the patients in the groups were not different (P=0.26). 

**Table 2:** Change trends of clinical presentation in the two groups during 6 weeks of follow-up

Week								Mean difference (95%CI) Placebo-Sucralfate	Interaction
		1 Median (Q1,Q3)	2 Median(Q1,Q3)	3 Median(Q1,Q3)	4 Median(Q1,Q3)	5 Median(Q1,Q3)	6 Median(Q1,Q3)		P-value
Rectal Bleeding	Sucralfate (n=24)	<0.001 (<0.001,1)	<0.001 (<0.001,0.5)	<0.001 (<0.001,<0.001)	<0.001 (<0.001,<0.001)	<0.001 (<0.001,<0.001)	<0.001 (<0.001,<0.001)	0.158 (0.017-0.353)	0.003
	Placebo (n=28)	<0.001 (<0.001,1)	<0.001 (<0.001,<0.001)	<0.001 (<0.001,1)	<0.001 (<0.001,1)	<0.001 (<0.001,1)	<0.001 (<0.001,1)		
Rectal Pain	Sucralfate (n=24)	1 (<0.001,1)	<0.001 (<0.001,1.5)	<0.001 (<0.001,1)	<0.001 (<0.001,1)	<0.001 (<0.001,1)	<0.001 (<0.001,<0.001)	0.377 (0.006-0.738)	<0.001
	Placebo (n=28)	<0.001 (<0.001,1)	<0.001 (<0.001,1.5)	<0.001 (<0.001,1)	1 (<00.1,2)	1 (<00.1,2)	1 (<00.1,2)		
Diarrhea	Sucralfate (n=24)	<0.001 (<0.001,1)	<0.001 (<0.001,1)	<0.001 (<0.001,1)	<0.001 (<0.001,1)	<0.001 (<0.001,1)	<0.001 (<0.001,1)	0.428 (0.181-0.676)	0.002
	Placebo (n=28)	<0.001 (<0.001,<0.001)	1 (<0.001,1)	1 (<0.001,1)	1 (1,1)	1 (0.5,2)	1 (1,1.5)		
Fecal urgency	Sucralfate (n=24)	<0.001 (<0.001,1)	<0.001 (<0.001,1)	<0.001 (<0.001,1)	<0.001 (<0.001,1)	<0.001 (<0.001,0.25)	<0.001 (<0.001,0.25)	0.16 (0.084-0.412)	0.002
	Placebo (n=28)	<0.001 (<0.001,<0.001)	<0.001 (<0.001,1)	<0.001 (<0.001,1)	1 (<0.001,1)	1 (<00.1,1)	1 (<00.1,1)		
Total	Sucralfate (n=24)	1 (<00.1,3)	1 (<00.1,3)	1 (<00.1,1)	1 (<00.1,2)	1 (<00.1,1.25)	<0.001 (<0.001,1)	1.11 (0.377-1.85)	<0.001
	Placebo (n=28)	0.5 (<00.1,2)	1 (1,3)	2 (1,3)	3 (2,4)	3 (2,4)	3 (2,4)		

**Table 3:** Change trends of RTOG Severity in the two groups during 6 weeks of follow-up

** Week**								**Mean difference (95%CI)** Placebo-Sucralfate	**Interaction**
		**1** Median(Q1,Q3)	**2** Median(Q1,Q3)	**3** Median(Q1,Q3)	**4** Median(Q1,Q3)	**5** Median(Q1,Q3)	**6** Median(Q1,Q3)		**P-value**
Diarrhea RTOG	Sucralfate (n=24)	<0.001 (<0.001,1)	<0.001 (<0.001,1)	<0.001 (<0.001,1)	<0.001 (<0.001,1)	<0.001 (<0.001,1)	<0.001 (<0.001,1)	0.641(0.149-1.132)	0.002
	Placebo (n=28)	<0.001 (<0.001,<0.001)	1 (<0.001,1)	1 (<0.001,1)	1 (1,1)	1 (0.5,2)	1 (1,1.5)		
Cystitis RTOG	Sucralfate (n=24)	1 (<0.001,1)	1 (<0.001,1)	1 (<0.001,1)	1 (1,1)	1 (0.75,1)	1 (<0.001,1)	0.171(-0.228 - 0.57)	0.27
	Placebo (n=28)	<0.001 (<0.001,1)	<0.001 (<0.001,1)	1 (<0.001,2)	1 (<0.001,2)	1 (<0.001,2)	1 (<0.001,2)		

## Discussion

This randomized study is a placebo-controlled, double-blind clinical trial which assessed the preventive effects of topical sucralfate 7% ointment at a dose of 1 g/twice a day in the prevention of ARP in pelvic cancer patients receiving radiotherapy. The results from this study show that sucralfate ointment is superior to the placebo in the prevention of the incidence of ARP and related symptoms in some aspects. Proctitis is a critical complication of radiotherapy in pelvic malignancies. The lower colon and the rectum are usually involved during pelvic radiotherapy, thus the patients may experience proctitis during or shortly after treatment (25-28). Alterations in bowel habits and diarrhea are the most frequent discomfort in these patients (28). Several pharmacotherapies have been tried to prevent or treat acute proctitis, but the results have not been satisfactory yet (4, 9-11). Topical preparations of sucralfate have been used successfully to heal wounds in several conditions, including radiation-induced mucositis and proctosigmoiditis, erythmatous radiation skin dermatitis, resistant peristomal and perianal excoriation, second and third degree burns, giant refractory solitary rectal ulcer syndrome, anal fistolotomy and skin excoriation around enterostomas (18, 19, 29-41). 

Henrikkson et al. evaluated the impact of oral sucralfate (as granule) on the prevention and therapy of acute bowel discomfort (6). They instructed the patients to use one package of sucralfate (1 g) in water every four hours. Our trial was aimed to assess the topical rectal product of sucralfate (e.g., 7% twice daily) which required fewer times of drug administration. The incidence of proctitis complications, which are mainly fecal urgency and diarrhea, were clearly reduced in the sucralfate group in comparison to the placebo group. With regards to fewer incidence of diarrhea in the sucralfate group, they would need less consumption of anti-diarrheal medicines including loperamide. One concern of use of loperamide is that, it paralyzes the smooth muscle to exert its effect,which may be dangerous since it causes fluid trapping in the bowls and increases the risk of bowel strangulation (42). Lower incidence of diarrhea in the sucralfate group would decrease the need of loperamide administration, resulting to less loperamide-related side effects. The results of our study show that sucralfate improved the quality of life of the patients during therapy, a finding that could be associated with the fewer incidences of adverse effects of radiotherapy with sucralfate compared to placebo. In previous studies, it has been shown that alleviating the radiation-induced bowel symptoms is associated with enhanced quality of life during radiotherapy. This increases their chance of completing the course of treatment (6, 12).

The exact mechanism of usefulness of sucralfate in proctitis in unknown but several explanations could be presented. Local synthesis of prostaglandin E2 at injured area is promoted by sucralfate, resulting into blood flow enhancement, surface migration of cell, and the resultant cytoprotective effect (34, 43, 44). Also, it has antimicrobial activity (45) and angiogenic effects (46). Histological examinations have shown that sucralfate stimulates formation of collagen, and vascular and granulation tissue (47). Sucralfate also enhances the binding of epidermal growth factor, and it has antioxidant effect which helps wound healing (48). The antioxidant effects of sucralfate may contribute to both the protection and healing of damaged mucus surfaces (49). Activation of both prostaglandin and nitric oxide also attributes to the cytoprotectivity of sucralfate (50). The presence of sucralfate in the rectum area could cause early adhesion to the induced damage (51) and diminish the incidence of rectal bleeding. 

In our study, sucralfate administration was associcated with less rectal pain. The pain reduction mechanism might be that it prohibits the release of inflammatory cytokines from the damaged cells (19). Moreover, sucralfate breaks down into aluminum salts and anionic sulfonate esters in the acidic pH of the rectum, the aluminum salts form aluminum-hydroxide molecules which neutralize bile acids and prevent them from worsening the wound (51). Sucralfate forms a protective barrier at the damaged site (52) which prevents further damage due to mechanical damage because of defecation and gastric enzymes. 

***Study limitations:*** Although this trial proposes positive results and it was conducted as a prospective placebo-controlled study, a cautious should be taken here, since we still do not know the optimal preventive period and the consequences of long-term use of topical sucralfate, exceeding the period of 6 weeks. To discuss the mechanisms by which sucralfate plays its roles, endoscopic evaluations would have been valuable (28). Nevertheless, because of the risk of unrepairable harm to the internal sphincter, which may cause incontinency, we did not find it ethical. Besides, since proctitis is an inflammatory reaction due to radiotherapy, any mechanical stimulation to the area should be avoided as it could worsen the condition. 

In conclusion the results of the present study suggest that sucralfate leads to fewer incidence of radiotherapy-induced acute proctitis with lower pain scores and higher scores of quality of life with no adverse effects. Considering all the promising results, it could be concluded that sucralfate may be beneficial in preventing radiotherapy-induced acute proctitis. 
